# Novel Methods in the Surveillance of Influenza-Like Illness in Germany Using Data From a Symptom Assessment App (Ada): Observational Case Study

**DOI:** 10.2196/26523

**Published:** 2021-11-04

**Authors:** Caoimhe Cawley, François Bergey, Alicia Mehl, Ashlee Finckh, Andreas Gilsdorf

**Affiliations:** 1 Ada Health GmbH Berlin Germany

**Keywords:** ILI, influenza, syndromic surveillance, participatory surveillance, digital surveillance, mobile phone

## Abstract

**Background:**

Participatory epidemiology is an emerging field harnessing consumer data entries of symptoms. The free app Ada allows users to enter the symptoms they are experiencing and applies a probabilistic reasoning model to provide a list of possible causes for these symptoms.

**Objective:**

The objective of our study is to explore the potential contribution of Ada data to syndromic surveillance by comparing symptoms of *influenza-like illness* (ILI) entered by Ada users in Germany with data from a national population-based reporting system called GrippeWeb.

**Methods:**

We extracted data for all assessments performed by Ada users in Germany over 3 seasons (2017/18, 2018/19, and 2019/20) and identified those with ILI (report of fever *with* cough *or* sore throat). The weekly proportion of assessments in which ILI was reported was calculated (overall and stratified by age group), standardized for the German population, and compared with trends in ILI rates reported by GrippeWeb using time series graphs, scatterplots, and Pearson correlation coefficient.

**Results:**

In total, 2.1 million Ada assessments (for any symptoms) were included. Within seasons and across age groups, the Ada data broadly replicated trends in estimated weekly ILI rates when compared with GrippeWeb data (Pearson correlation—2017-18: *r*=0.86, 95% CI 0.76-0.92; *P*<.001; 2018-19: *r*=0.90, 95% CI 0.84-0.94; *P*<.001; 2019-20: *r*=0.64, 95% CI 0.44-0.78; *P*<.001). However, there were differences in the exact timing and nature of the epidemic curves between years.

**Conclusions:**

With careful interpretation, Ada data could contribute to identifying broad ILI trends in countries without existing population-based monitoring systems or to the syndromic surveillance of symptoms not covered by existing systems.

## Introduction

### Background

Influenza is a disease that causes considerable morbidity and mortality each year [1] and has been the subject of research investigating the application of novel surveillance tools, including the potential use of data from web-based sources [2]. In many European countries, data on the syndromic surveillance of *influenza-like illness* (ILI) are collected via internet-based reporting tools run by national public health institutes [3]. In Germany, one such tool (*GrippeWeb*) collects data from voluntary participants, who are prompted to report, on a weekly basis, whether they have experienced any symptoms of an acute respiratory infection [4]. Such a tool is complementary to physician- and laboratory-based surveillance and helps to capture data from a population who have not or may not come into contact with the health care system, thus potentially providing a fuller picture of disease incidence within the population. Such population-based reporting tools might confer particular benefits during an epidemic or pandemic, if patterns of health care–seeking behavior change because of individuals’ reluctance or inability to visit doctors or clinics [5].

### Objectives

A growing number of studies have explored the potential contribution of additional web-based data sources to the surveillance of infectious diseases, including the aggregation of data from web-based newsfeeds [6,7] and analyses of Google search query data [8,9]. One additional possible source of data is from health-related smartphone apps. One such app, the symptom assessment tool Ada [10,11], collects basic demographic information and self-reported symptoms from users to suggest conditions that they may be experiencing. In this study, we compare ILI symptoms reported by Ada users in Germany with ILI symptom data reported to GrippeWeb to explore the potential contribution of data from the Ada app to syndromic surveillance.

## Methods

### Description of Ada

The symptom assessment app Ada can be downloaded and used free of charge. Users must declare that they are aged ≥16 years in order to create an account; however, account owners can assess symptoms on behalf of others, including those aged <16 years. Users provided their age, sex, and some basic medical history information before starting a *symptom assessment*, which begins with the question “*Let’s start with the symptom that’s troubling you the most*,” followed by “*Do you have any other symptoms?*” Symptoms are initially entered into a free-text field, with users selecting the best fit from a list of medically curated terms. On the basis of the initially entered symptoms and other user-provided information, including age and sex, a probabilistic reasoning model determines which additional questions to ask (ie, the exact set of symptoms asked about varies from assessment to assessment). At the end of an assessment, users are provided with a list of up to 5 possible causes for their symptoms, as well as advice on possible next steps, for example, whether the condition could be managed at home or whether consulting a physician or hospital is recommended. The Ada app can assess an extensive range of symptoms and conditions covering various medical specialties, not only those related to respiratory illness.

### Extraction of Ada Data and Definition of ILI

Data from all Ada assessments (ie, for any symptoms or complaints) completed by users in Germany between calendar week 27, 2017, and calendar week 26, 2020, were extracted; users may have completed only one or more than one assessment over this period. Users were classified as having ILI if they reported fever with *either* cough *or* sore throat (same ILI definition as used by GrippeWeb), either as initially entered symptoms (ie, users entered these terms directly or selected them from a dropdown list at the start of the symptom assessment), or in response to questions asked during an assessment. Questions were of the form: *“Do you have symptom x?*” where “*x” was fever, cough, or sore throat*.” Answer options were *yes*, *no*, or *I don’t know*; only *yes* responses were used. For fever, users were additionally asked to state what temperature their fever was, or to state *I don’t know*. *Yes* responses for fever were still used even if the users reported that they did not know what their temperature was.

### Description of GrippeWeb

The GrippeWeb system has been described in detail elsewhere [4]. Briefly, participants who registered were asked to log in on a weekly basis and report if they had experienced any of the main symptoms of a new respiratory illness (any cough, head cold, sore throat, or fever) in the preceding week (retrospective reporting for up to 4 weeks is also possible). Participants were asked to respond even if they had not experienced any symptoms. To reduce the bias possibly introduced by people reporting only during weeks when they are ill, participants must report to the GrippeWeb system at least 5 times to be included in data analyses. Participants who reported less than 10 times, but who met the definition for an acute respiratory infection (report of fever, cough, or sore throat) on 50% or more of these occasions were also excluded from data analyses [4]. The minimum age for participation in GrippeWeb is 14 years; however, parents can report on behalf of children aged <14 years.

### Calculation of Ada ILI Rates and Extraction of GrippeWeb ILI Rates

From calendar week 27, 2017 to calendar week 26, 2020 (ie, covering 3 flu seasons: 2017/18, 2018/19, and 2019/20), weekly raw Ada ILI rates were calculated by taking the number of assessments in which users met the definition of ILI, divided by the total number of assessments completed in Germany during that week. To account for differences between the age and sex of Ada users compared with the general population, the raw Ada rates were standardized (by age and sex) for the German population. German population size estimates were extracted from the United Nations Department of Economic and Social Affairs website [12]. Weekly standardized ILI rates were also stratified across 5 age groups (0-4, 5-14, 15-34, 35-59, and 60 years) by calculating 3-weekly smoothed averages and taking median values from the 3 seasons for each age group (2017/18, 2018/19, and 2019/20).

Weekly population-adjusted GrippeWeb ILI estimates for calendar week 27, 2017 to calendar week 26, 2020, were extracted manually from reports published on the GrippeWeb website [13]. This was done using a linear regression equation that predicted y-axis values (the population-adjusted ILI rates) for each calendar week. Using the same method, weekly age-stratified ILI estimates were extracted for the same age groups mentioned above, on the basis of data from the 2011/12 to 2016/17 seasons (three-weekly smoothed averages; median values from the 6 seasons), as reported in [4].

### Data Analyses

We plotted Ada population-adjusted ILI rates alongside GrippeWeb rates in time series plots: overall and stratified by age. Owing to differing denominators, it should be noted that actual ILI rates were not directly comparable between the 2 data sources; in GrippeWeb, participants were prompted to report to the system each week regardless of whether they had symptoms, that is, the denominator included those with and without ILI symptoms. In contrast, in Ada, users only consulted and reported to the tool when they had symptoms. Furthermore, in addition to these symptomatic ILI users, the Ada denominator also included users who completed an assessment for any type of symptom, including those unrelated to respiratory illness.

The nature of the relationship between Ada and GrippeWeb data was explored using scatterplots, and correlations were explored using Pearson correlation coefficient—values for *r*, 95% CI, and *P* values at the significance level of .05. All plots and analyses were performed using Excel (Microsoft Inc) and Stata (StataCorp) version 11.

### Ethics and Data Privacy

We analyzed pseudonymized health data for public health purposes according to the European General Data Protection Regulation. Ada users were duly informed of the use of their data (information available at any time in Ada’s privacy policy). In addition, users maintained their right to object to such processing for reasons arising from their particular situation, as required by the General Data Protection Regulation. Raw numbers presented in this study are rounded to the nearest 10 for data privacy reasons.

## Results

### Description of Ada User Population

In total, 2,108,110 assessments (for any symptoms) performed by Ada users in Germany between calendar week 27, 2017, and calendar week 26, 2020, were analyzed. The quantity of data available for analysis varied over time for several reasons. These include the following: (1) user numbers can change from month to month depending on marketing activities and (2) owing to changes in Ada’s data privacy and use policy, data for only a restricted subset of users were available for the period May 2018 to November 2019.

Table 1 provides an overview of the number of assessments completed per season and the demographic characteristics of Ada users. The median number of assessments completed per week was lower in the 2017/18 season than in 2018/19 and 2019/20, and the IQR was greater in the 2017/18 and 2019/20 seasons than in the 2018/19 seasons. Overall (over all seasons), a large majority of users were female (1,470,740/2,108,110, 69.77%) and aged between 15 and 34 years (1,556,490/2,108,110, 73.83%), with fewest users in the youngest age group (0-4 years: 18,150/2,108,110, 0.86%), followed by the oldest age group (>60 years: 49,980/2,108,110, 2.37%). There were fewer users in the age group of 5-14 years in seasons 2018/19 and 2019/20 than those in the 2017/18 seasons, most likely because of a change in the minimum sign up age from 13 to 16 years in May 2018. In total (over all seasons), 2.24% (47,300/2,108,110) of Ada users reported ILI. This proportion was slightly higher among male users (2.8%) than among female users (2%), and also varied by week and age group (see sections below on comparison of ILI rates).

**Table 1:** Number of assessments completed and demographics of Ada users in Germany, seasons 2017/18, 2018/19, and 2019/20^a^.

Demographics			Season				
			2017/18		2018/19		2019/20
Total number assessments completed			565,880		625,130		917,100
Number assessments per week, median (IQR)			10,240 (400-15,240)		12,020 (10,840-13,310)		16,950 (14,750-19,970)
**Sex, n (%)**							
	Female	389,700 (68.87)		442,490 (70.78)		638,550 (69.62)	
	Male	176,190 (31.14)		182,640 (29.21)		278,540 (30.37)	
**Age, n (%)**							
	0-4	4100 (0.72)		6070 (0.97)		7980 (0.87)	
	5-14	44,480 (7.86)		18,620 (2.97)		13,810 (1.51)	
	15-34	400,220 (70.73)		464,460 (74.3)		691,810 (75.43)	
	35-59	92,640 (16.37)		108,500 (17.36)		161,470 (17.6)	
	≥60	11,000 (1.94)		15,560 (2.49)		23,420 (2.55)	

^a^A season begins in calendar week 27 of any given year and finishes at the end of calendar week 26 of the following year. Numbers rounded to the nearest 10 for data privacy reasons

### Description of GrippeWeb Users

Briefly, the number of reports on the GrippeWeb system has increased from approximately 800 per week in mid-2011 [4] to approximately 5000 per week in 2020 [14]. In 2017, 56% of participants were females and the sample represented the German population quite well across age groups, albeit with some overrepresentation of those aged 40-59 years and some underrepresentation of those aged 15-24 years and 60 years [4].

### Comparison of Population-Adjusted ILI Rates Across Three Seasons

**Figure 1:**
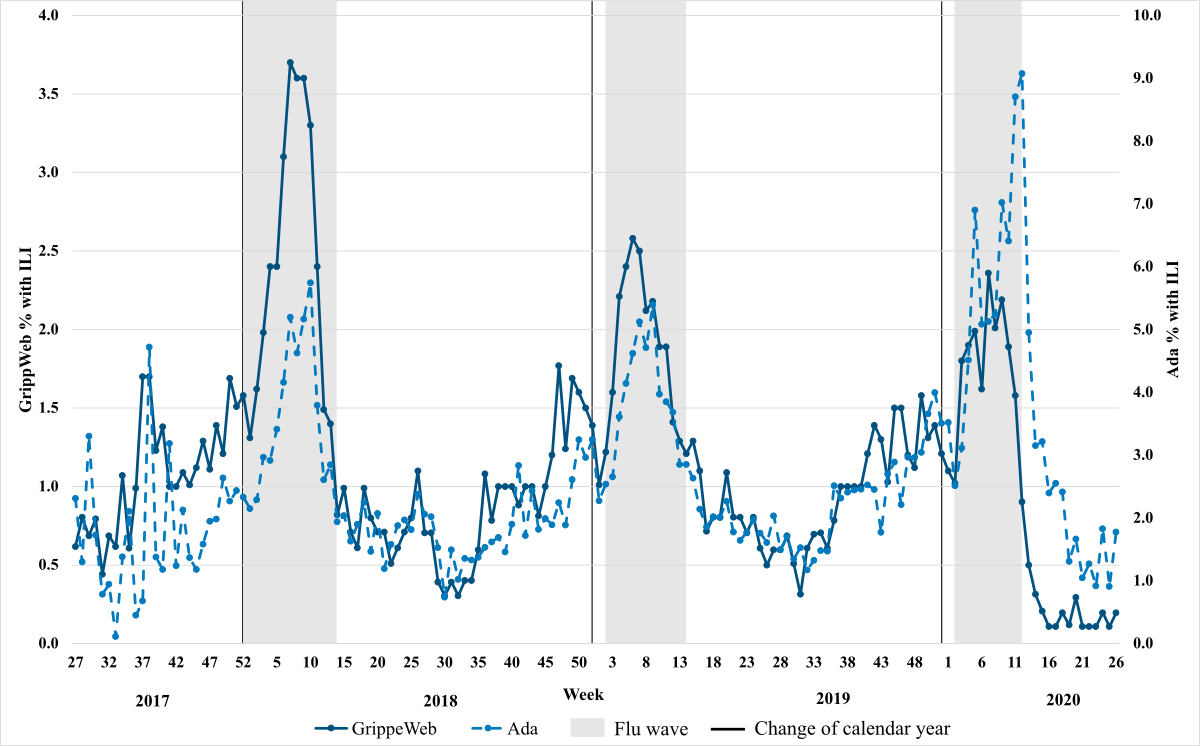
Weekly population-adjusted influenza-like illness rates in Germany as estimated by GrippeWeb (solid line) and Ada (dashed line), calendar week 27, 2017 to calendar week 26, 2020. GrippeWeb data extracted from the report by Buchholz et al [13]. The flu wave period is defined each year by the Robert Koch Institute according to virological surveillance. ILI: influenza-like illness.

Figure 1 shows the weekly population-adjusted ILI rates estimated using Ada and GrippeWeb data for seasons 2017/18, 2018/19, and 2019/20 (recall that actual rates for Ada and GrippeWeb are not directly comparable and that we rather sought to compare trends in rates). The figure shows that within seasons, trends were broadly similar between the 2 data sources, albeit with specific differences. For example, although both data sources showed the start of the peak ILI season around the same time each year (with increases from week 2 in 2017/18 and 2018/19 and from week 3 in 2019/20), there were differences in the exact nature and timing of the peaks. In 2017/18, the GrippeWeb peak ILI rate was recorded in week 7, whereas the Ada peak was recorded in week 10. In 2018/19, peak ILI rates were recorded by GrippeWeb and Ada in weeks 6 and 9, respectively, and in 2019/20 in weeks 7 and 12, respectively. Results of Pearson correlation indicated that the Ada and GrippeWeb data were significantly correlated, with *P*<.001 in all 3 seasons. The correlation coefficient *r* for seasons 2017/18, 2018/19 and 2019/20 was 0.86 (95% CI 0.76-0.92), 0.90 (95% CI 0.84-0.94), and 0.64 (95% CI 0.44-0.78), respectively. Scatterplots of the population-adjusted Ada and GrippeWeb ILI rates for each individual season are shown in Multimedia Appendices 1-3.

Looking at Figure 1 and comparing across seasons within a single data source, the GrippeWeb data showed higher peak ILI rates in 2017/18 compared with 2018/19 or 2019/20 (with the relative height of the *waves* being similar during these latter 2 seasons). This pattern was not seen in the Ada data, where the relative height of the waves was similar in 2017/18 and 2018/19, but higher in 2019/20 (with particularly high rates seen in weeks 11 and 12 of 2020). In 2020, both data sources showed a steep decline in ILI rates after week 11 (GrippeWeb) or week 12 (Ada). Between weeks 12 and 26 of 2020, ILI rates were low in both data sources; however, this trend was seen particularly in the GrippeWeb data when compared with corresponding weeks in previous years. ILI rates in the Ada data over these weeks in 2020 were slightly lower but broadly similar to those seen in previous years.

### Comparison of Age-Stratified Population-Adjusted ILI Rates

**Figure 2:**
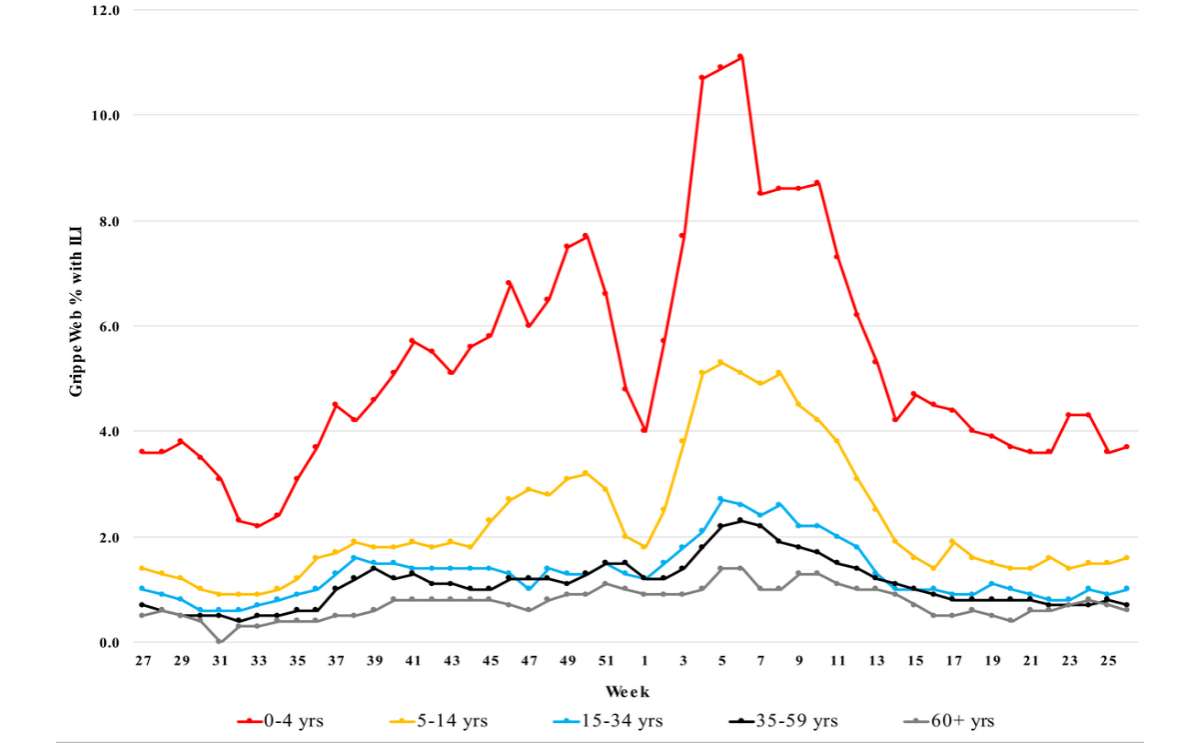
Population-adjusted age-stratified influenza-like illness rates as estimated by GrippeWeb (3 weekly moving averages, graphs show median values from 6 seasons 2011/12 to 2016/17). Data extracted from the study by Buchholz et al [4], as described in the Methods section. ILI: influenza-like illness.

**Figure 3:**
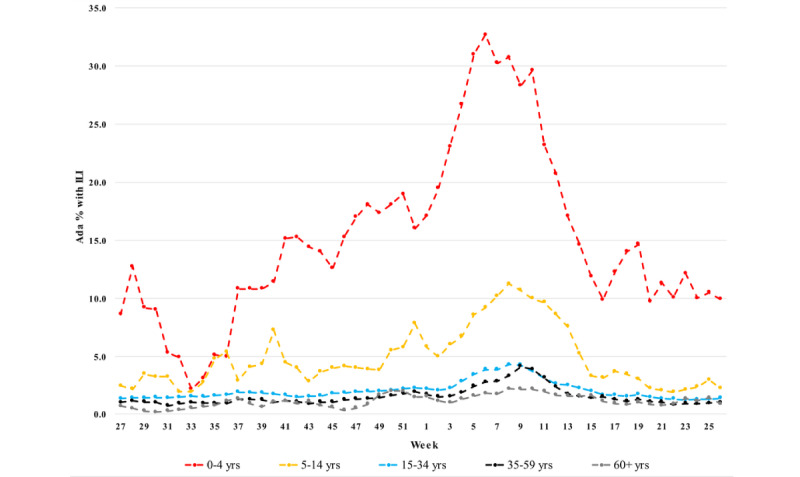
Population-adjusted age-stratified influenza-like illness rates as estimated by Ada (3 weekly moving averages, graphs show median values from 3 seasons 2017/18 to 2019/20). ILI: influenza-like illness.

Figures 2 and 3 show the age-stratified population-adjusted ILI rates estimated by GrippeWeb (median values from 6 seasons) and Ada (median values from 3 seasons), respectively. Across the 5 age groups, broad patterns were similar between the 2 data sources, with both showing the highest ILI rates among the youngest individuals (aged 0-4 and 5-14 years), with decreasing ILI rates with increasing age; the lowest ILI rates were observed among those aged 60 years in both data sources. Results of Pearson correlation for each age group indicated that the Ada and GrippeWeb data were significantly correlated, with *P*<.001 in all cases. The respective correlation coefficients for age groups 0-4, 5-14, 15-34, 35-59, and ≥60 were 0.93 (95% CI 0.88-0.96), 0.83 (95% CI 0.72-0.90), 0.89 (95% CI 0.82-0.94), 0.79 (95% CI 0.66-0.88), and 0.78 (95% CI 0.64-0.87). Scatterplots of the age-stratified ILI rates estimated by GrippeWeb and Ada are shown in Multimedia Appendices 4-8.

## Discussion

### Principal Findings

In this analysis, we have shown that within seasons and across age groups, the Ada data broadly replicated trends in estimated weekly ILI rates for Germany when compared with data from GrippeWeb, with the latter system having previously been shown to correspond well with other sources of national influenza surveillance data [4,15]. This broad congruence is encouraging, particularly given the very different nature of the tools and the way in which they collect data, and points to the potential value of data from a tool such as Ada.

In addition to broad trends, however, the specific nature and timing of an epidemic curve are likely to be of interest to health service providers as they plan health care resources [16]. Comparing the data presented in this paper with data presented in the annual influenza surveillance reports for Germany, in 2 out of 3 seasons analyzed (2017/18 and 2018/19), GrippeWeb showed peak ILI rates a week earlier than was seen in health care system-based surveillance data [17,18]. This might be explained by a time lag between the onset of symptoms and the point at which an individual visits a physician (and the physician can notify the health authorities), and demonstrates the potential of web-based reporting systems to detect the start of epidemics earlier than traditional systems. In all 3 seasons, the timing of Ada’s peak ILI rates corresponded with weeks during which the influenza epidemic was also reported to have peaked in national surveillance data. This overlap was encouraging, but in this retrospective analysis, Ada did not demonstrate the ability for early epidemic detection. That said, as Ada data can provide symptom trends in real time (without the time lag typical of national surveillance data), future analyses could explore a possible time benefit by prospectively comparing daily notification data with Ada data.

When comparing trends across seasons within an individual data source, GrippeWeb showed the highest ILI peaks in 2017/18, which was also reported in national surveillance data to be a particularly bad flu season in Germany, with less severe epidemics reported in 2018/19 and 2019/20 [14,18]. Ada detected the highest ILI rates in 2019/20, with lower intensity ILI peaks in 2017/18 and 2018/19. Various hypotheses have been suggested for this anomaly.

As Ada users consult the app when desired, the data are subject to variability, depending on how many users consult the app and for what symptoms in any given week (and this may in turn be influenced by marketing activities or other factors, such as public interest). An examination of trends in the symptoms initially entered into the app between January and April 2020 revealed that between March 12 and March 24, 2020 (covering dates in weeks 10, 11, and 12), there was a sharp increase in the proportion of initially entered symptoms, which were fever, cough, or sore throat. National influenza surveillance data showed that the influenza epidemic in Germany came to an end around this time [19]. However, this period corresponds with weeks when high SARS-CoV-2 case numbers were reported in Germany during the spring 2020 wave of the COVID-19 pandemic [20], and also with weeks when various COVID-19 control measures were introduced by the German government (eg, recommendations for social distancing and the closure of schools) [21].

It is possible that the sharp increase in the proportion of user-entered symptoms, which were fever, cough, or sore throat, was due to the detection of COVID-19 cases. However, sentinel surveillance data showed that the proportion of samples positive for SARS-CoV-2 in weeks 10, 11, and 12 was much lower (between approximately 0.5% and 1.5%) than the proportion positive for influenza viruses (between approximately 20% and 40%) [19]. Therefore, it seems unlikely that Ada ILI rates possibly caused by SARS-CoV-2 would have been higher than ILI rates caused by influenza (or other viruses). An alternative, more probable explanation is that users were more likely to consult Ada for ILI symptoms during these specific weeks because of heightened awareness or concern regarding their symptoms, as a result of the prevailing COVID-19 pandemic. Between weeks 12 and 26 of 2020, Ada ILI rates were slightly lower but broadly similar to those reported over corresponding weeks in previous years, whereas GrippeWeb rates were considerably lower in comparison to corresponding weeks in previous years. Low incidence of influenza was reported in the weeks following the start of the COVID-19 pandemic in March 2020 (and also over the winter 2020/21 season), and is thought to be an effect of nonpharmaceutical pandemic control measures (recommendations for social distancing, intermittent closure of schools and kindergartens, closure of shops and restaurants, etc). This reduction in ILI rates was only partially detected in the Ada data.

Other possible explanations for the high ILI peak seen in the Ada data in weeks 11 and 12 of 2020 include the possibility that a routine update to Ada’s medical model around this time influenced the frequency with which questions related to ILI were asked. However, our examination of the nature of the change made indicates that this is likely to have had a much lesser impact than the user-driven changes that we observed when examining only the initially entered symptoms, which were unaffected by changes to the medical model.

Owing to a change in Ada’s privacy policy, data for only a subset of users were available for the period May 2018 to November 2019, and it is possible that differences or biases between these users and those who consulted the app before or after this period contributed to some of the differences observed. In 2017/18, national sentinel surveillance data showed that ILI consultation rates among those aged 35-59 years were particularly high [17]. Given the predominance of young individuals aged 15-34 years in the Ada data and the smaller proportion of those aged 35 years, it is possible that our sample of users aged 35 years was not representative of adults in this age group, or that older age groups used the app in a different way to younger ones (eg, possibly being less concerned about or less likely to consult the app for ILI symptoms). These hypotheses might provide a partial explanation for the lower Ada ILI peak in 2017/18 compared with later seasons (ie, Ada may not have captured a possible higher ILI incidence among older age groups in the 2017/18 season).

### Limitations

The limitations of the Ada data include that users are predominantly young and female, and that the data may be subject to fluctuations resulting from user behavior, marketing activities, or changes to Ada’s medical models. For these reasons, the data must be interpreted with caution. The strengths of the Ada data include that they are real time and cover a broad range of symptoms. Their primary value may lie in providing initial information on broad ILI trends for countries without existing population-based monitoring systems or information on other symptoms not covered by existing syndromic surveillance systems, including those with noninfectious causes (eg, relating to the effects of pollution, food allergies, or other common conditions such as migraine). Further studies should validate the potential of tools such as Ada for the future of syndromic surveillance, making comparisons also to laboratory-confirmed surveillance data. The advantages of app-based systems include the rapid collection of data from a large pool of individuals. Tools that collect data in a standardized and systematic way (eg, the COVID Symptom Study [22]) could make rapid and impactful contributions, particularly during a pandemic.

## Appendix

Multimedia Appendix 1 Scatterplot of weekly population adjusted GrippeWeb and Ada influenza-like illness rates for the period from week 27, 2017 to week 26, 2018.

Multimedia Appendix 2 Scatterplot of weekly population adjusted GrippeWeb and Ada influenza-like illness rates for the period from week 27, 2018 to week 26, 2019.

Multimedia Appendix 3 Scatterplot of weekly population adjusted GrippeWeb and Ada influenza-like illness rates for the period from week 27, 2019 to week 26, 2020.

Multimedia Appendix 4 Scatterplot of weekly population-adjusted influenza-like illness rates among those aged 0-4 years. GrippeWeb data points represent median weekly values over six seasons (2011/12–2016/17), and Ada data points represent median weekly values over three seasons (2011/12–2016/17).

Multimedia Appendix 5 Scatterplot of weekly population-adjusted influenza-like illness rates among those aged 5-14 years. GrippeWeb data points represent median weekly values over six seasons (2011/12–2016/17), and Ada data points represent median weekly values over three seasons (2011/12–2016/17).

Multimedia Appendix 6 Scatterplot of weekly population-adjusted influenza-like illness rates among those aged 15-34 years. GrippeWeb data points represent median weekly values over six seasons (2011/12–2016/17), and Ada data points represent median weekly values over three seasons (2011/12–2016/17).

Multimedia Appendix 7 Scatterplot of weekly population-adjusted influenza-like illness rates among those aged 35-59 years. GrippeWeb data points represent median weekly values over six seasons (2011/12–2016/17), and Ada data points represent median weekly values over three seasons (2011/12–2016/17).

Multimedia Appendix 8 Scatterplot of weekly population-adjusted influenza-like illness rates among those aged 60+ years GrippeWeb data points represent median weekly values over six seasons (2011/12–2016/17), and Ada data points represent median weekly values over three seasons (2011/12–2016/17).

## Abbreviations

ILI: influenza-like illness
