# Supplementary figures and images for: Novel Methods in the Surveillance of Influenza-Like Illness in Germany Using Data From a Symptom Assessment App (Ada): Observational Case Study
Source: JMIR Public Health Surveill. 2021 Nov 4;7(11):e26523. doi: 10.2196/26523 (PMC8722671; doi:10.2196/26523)

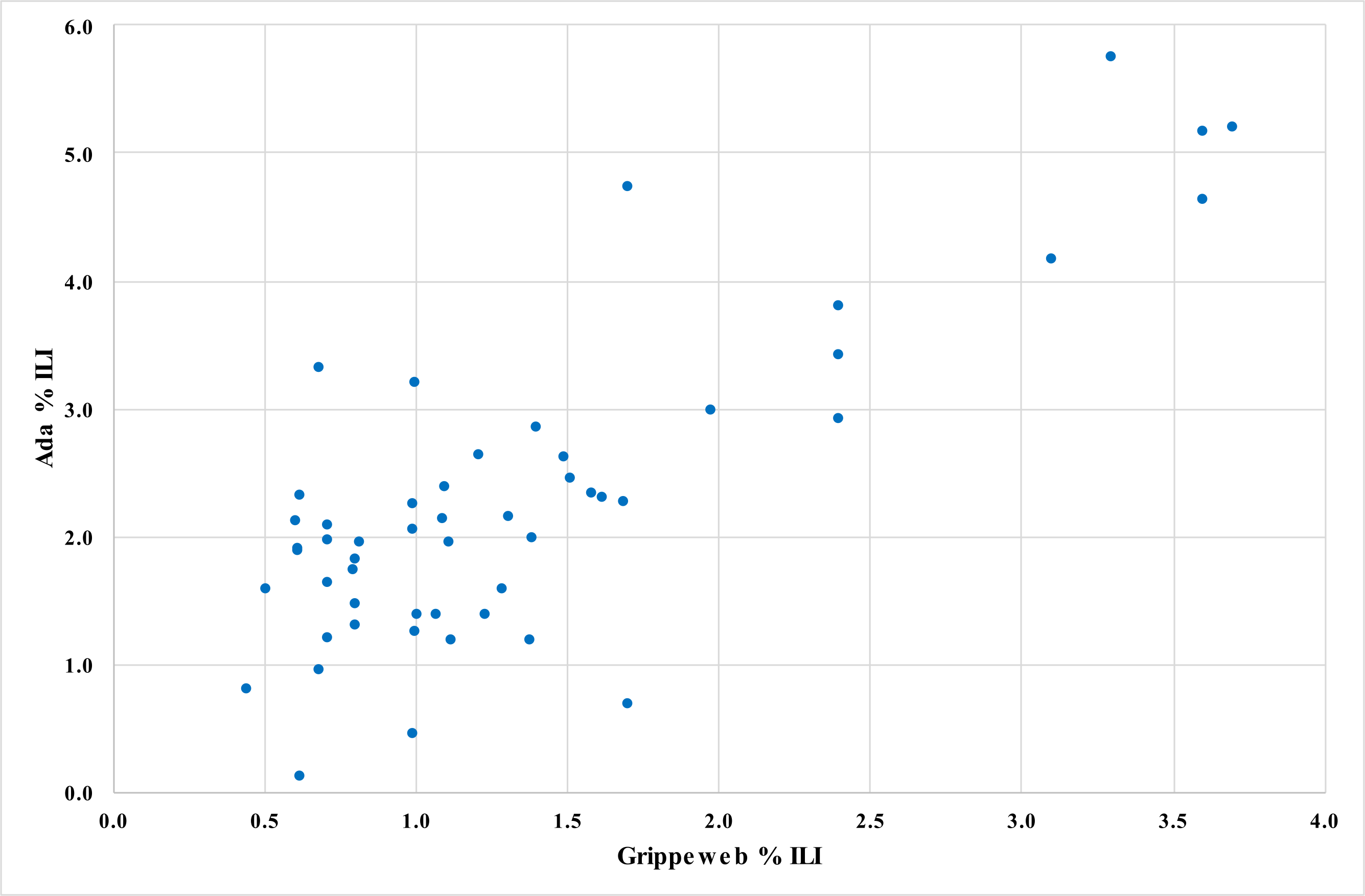

Supplement: Multimedia Appendix 1 [file publichealth_v7i11e26523_app1.png]

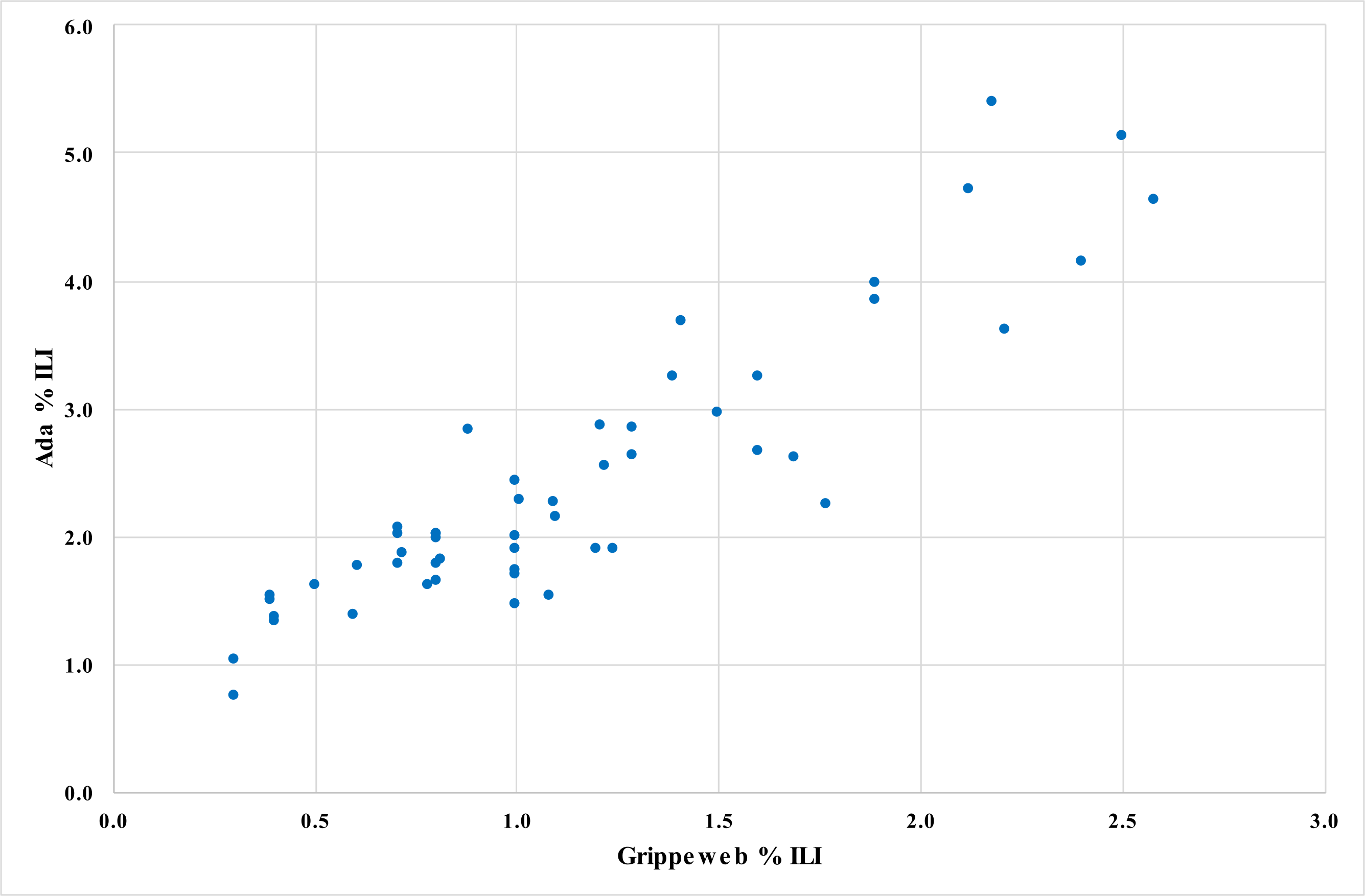

Supplement: Multimedia Appendix 2 [file publichealth_v7i11e26523_app2.png]

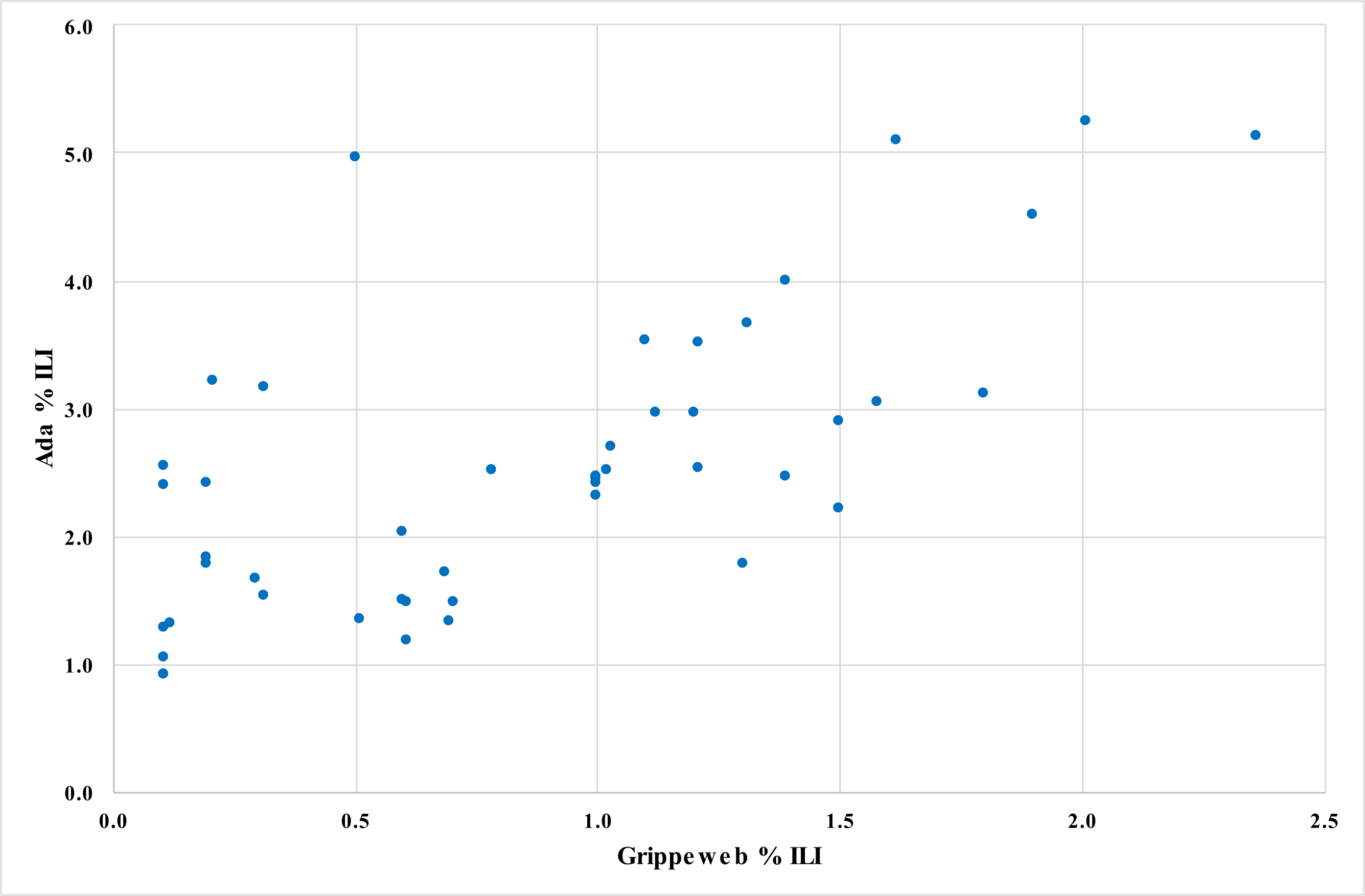

Supplement: Multimedia Appendix 3 [file publichealth_v7i11e26523_app3.png]

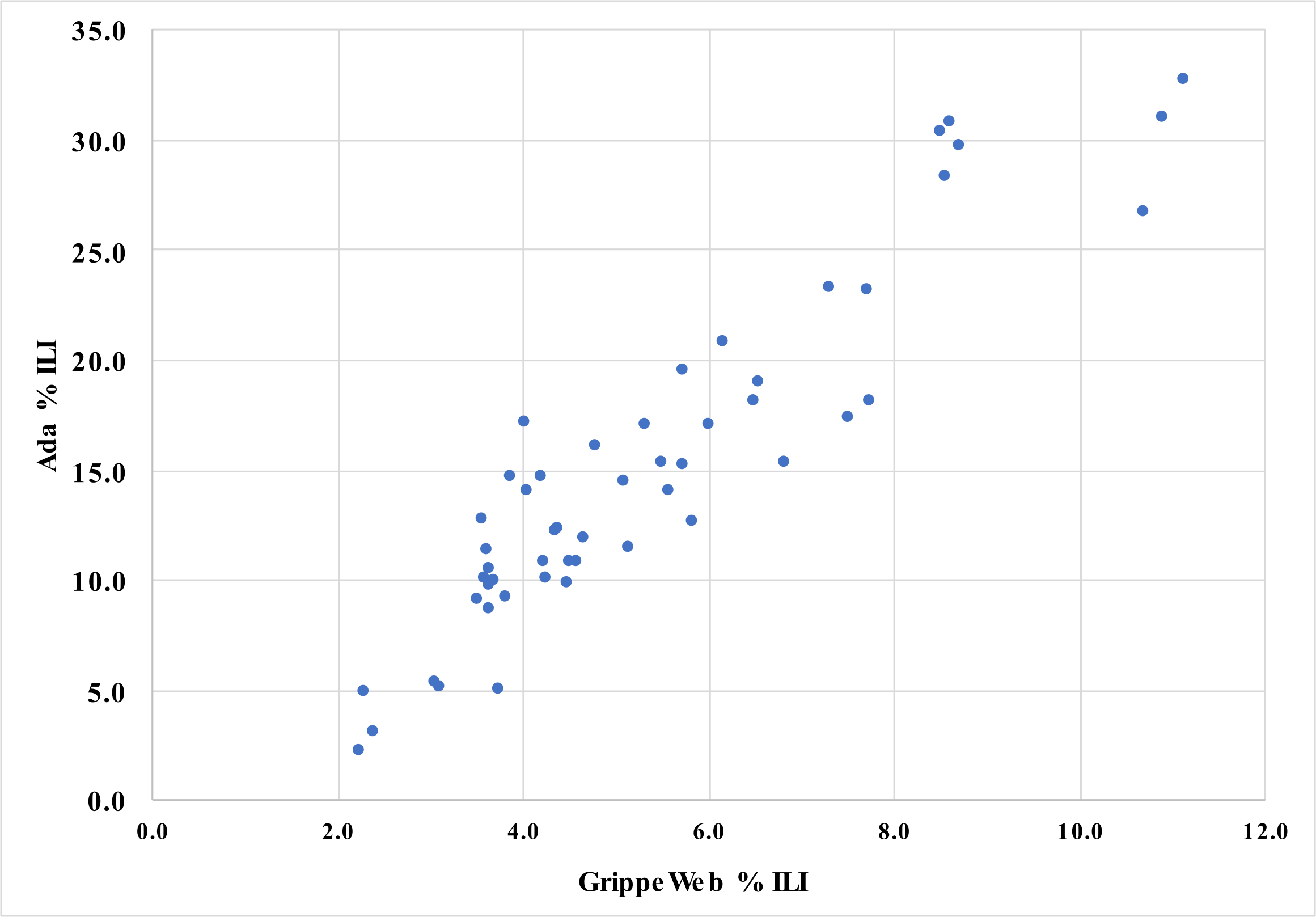

Supplement: Multimedia Appendix 4 [file publichealth_v7i11e26523_app4.png]

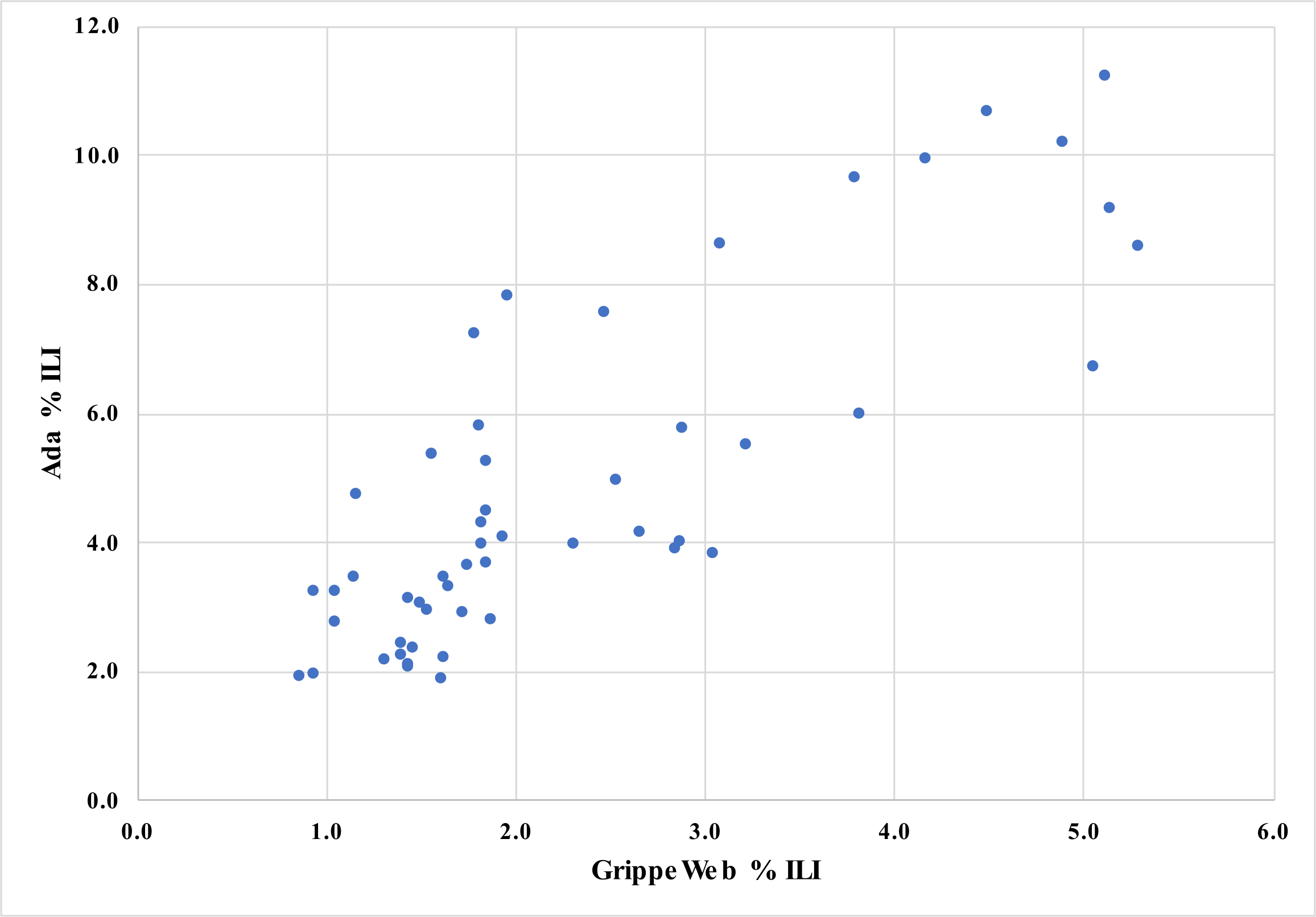

Supplement: Multimedia Appendix 5 [file publichealth_v7i11e26523_app5.png]

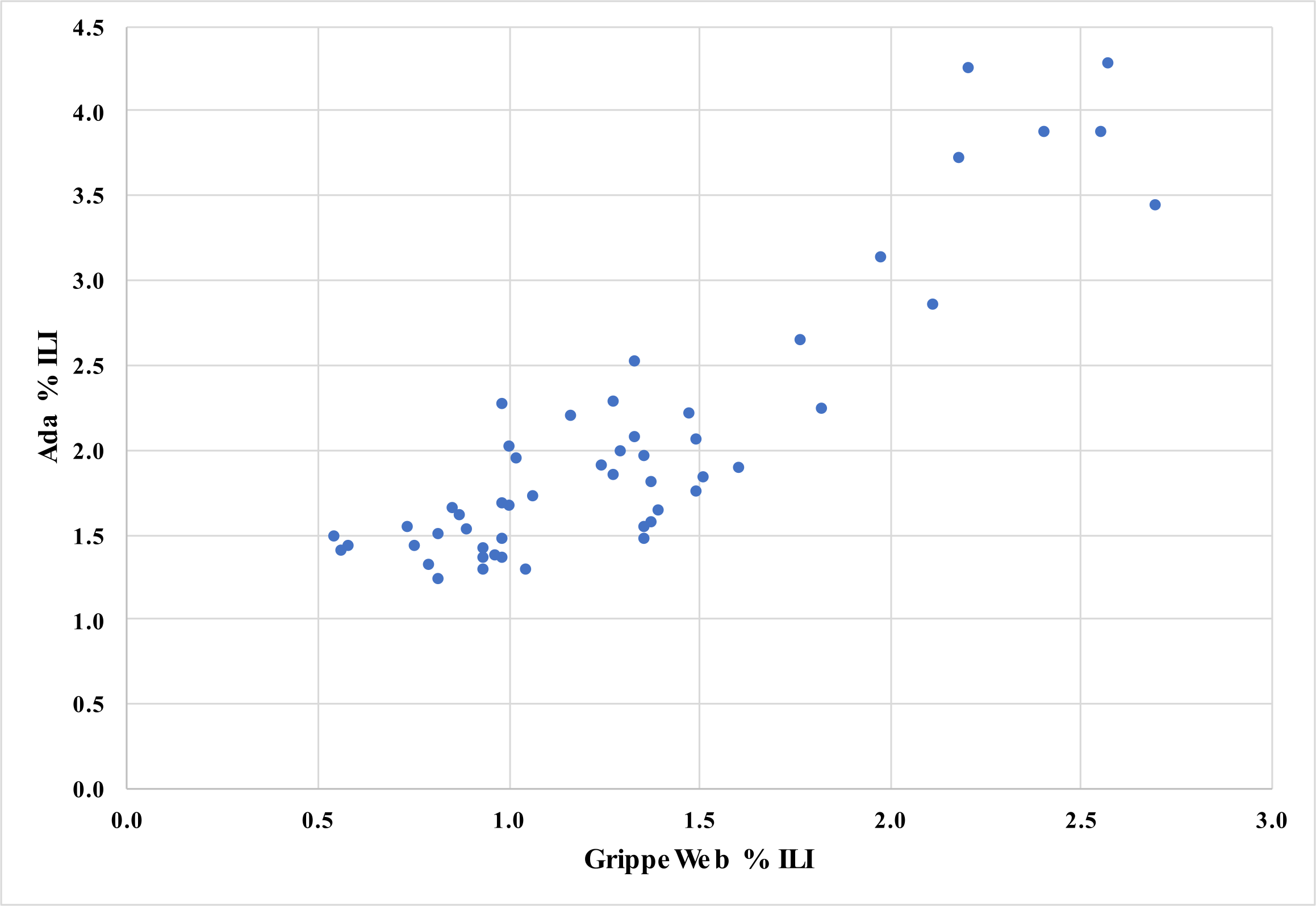

Supplement: Multimedia Appendix 6 [file publichealth_v7i11e26523_app6.png]

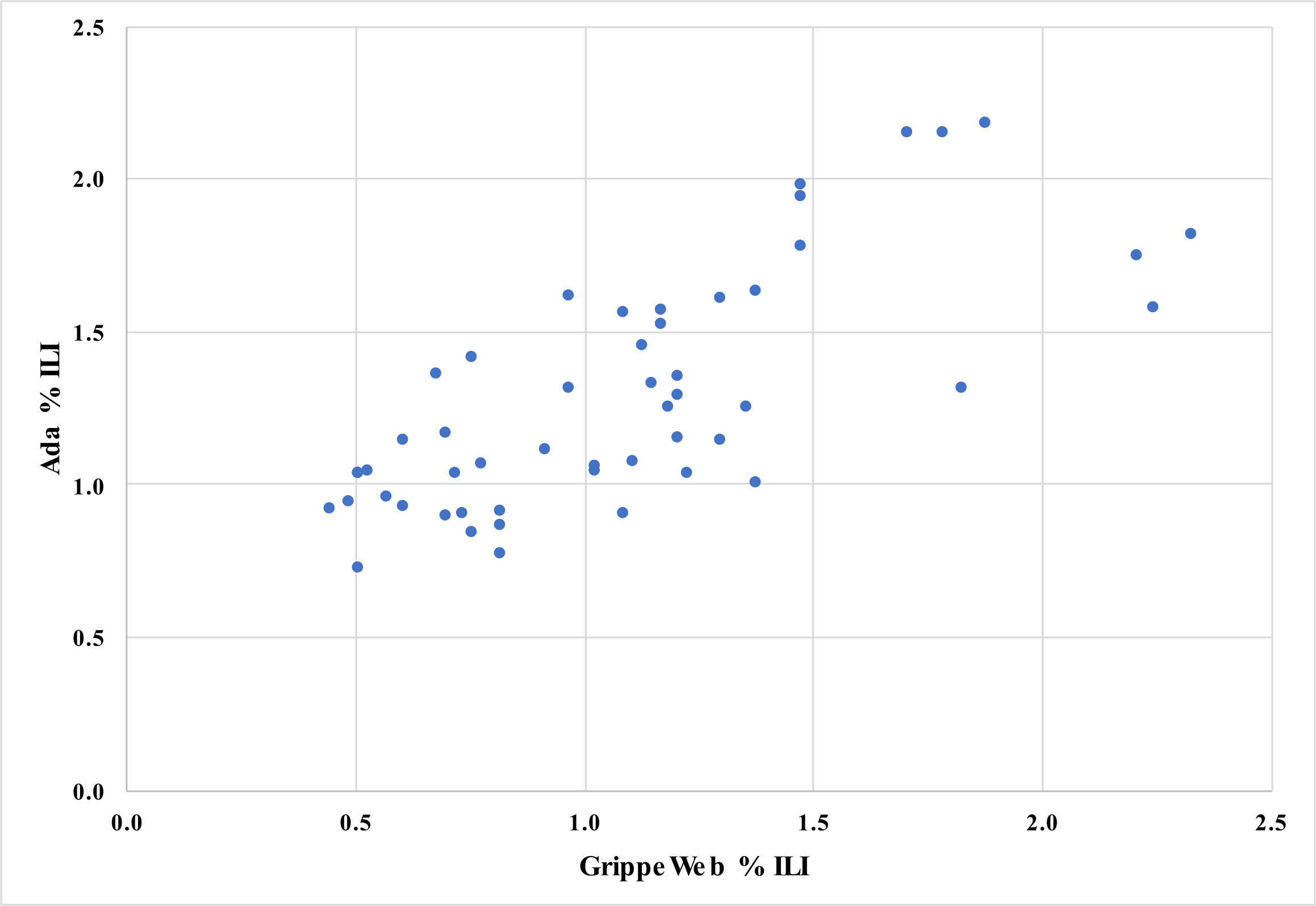

Supplement: Multimedia Appendix 7 [file publichealth_v7i11e26523_app7.png]

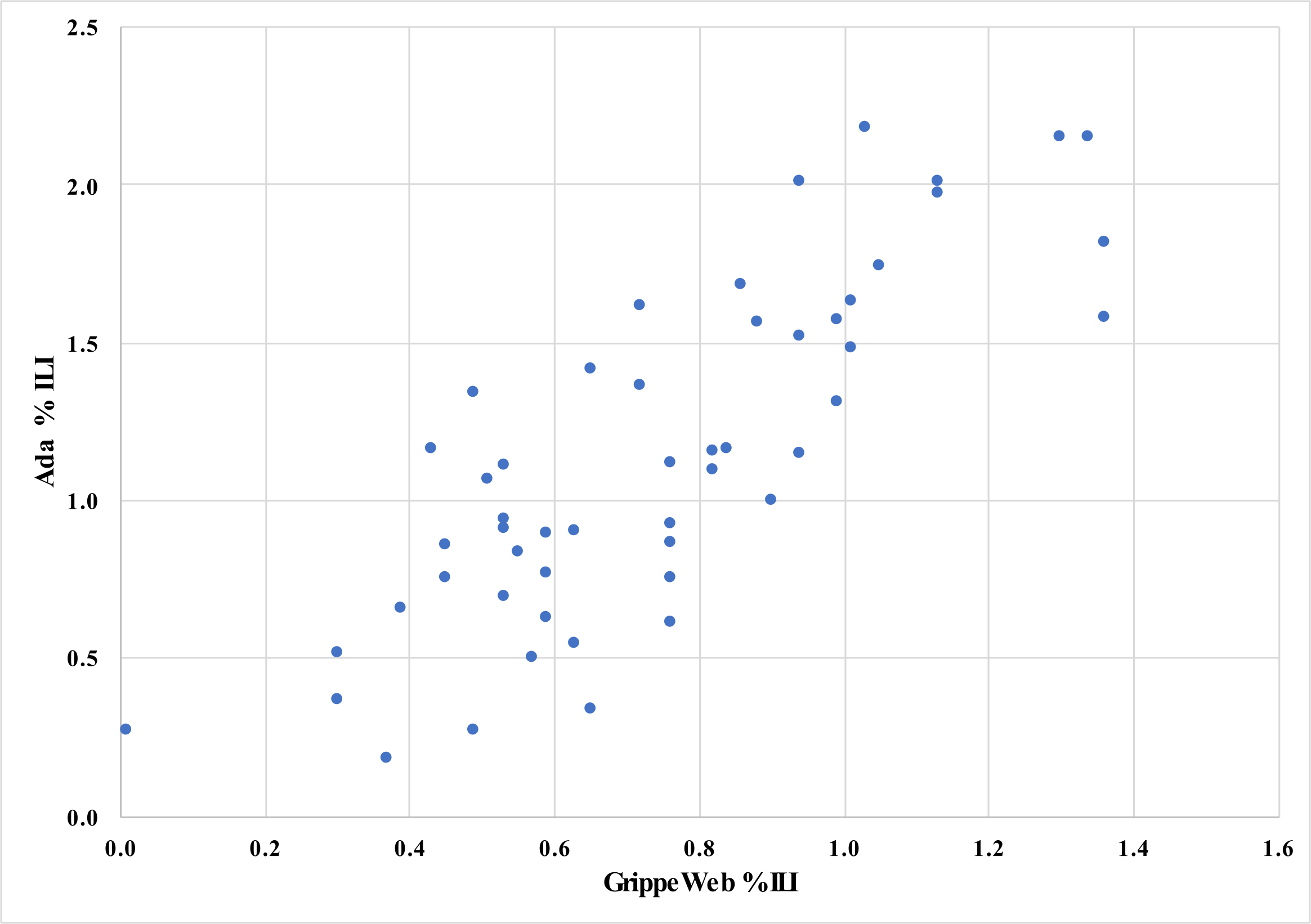

Supplement: Multimedia Appendix 8 [file publichealth_v7i11e26523_app8.png]
